# Association Among Cognition, Frailty, and Falls and Self‐Reported Incident Fractures: Results From the Canadian Longitudinal Study on Aging (CLSA)

**DOI:** 10.1002/jbm4.10679

**Published:** 2022-09-28

**Authors:** Ahreum Lee, Caitlin McArthur, George Ioannidis, Jonathan D. Adachi, Lauren E. Griffith, Lehana Thabane, Lora Giangregorio, Suzanne N. Morin, William D. Leslie, Justin Lee, Alexandra Papaioannou

**Affiliations:** ^1^ GERAS Centre for Aging Research Hamilton ON Canada; ^2^ Department of Health Research Methods, Evidence, and Impact McMaster University Hamilton ON Canada; ^3^ School of Physiotherapy Dalhousie University Halifax NS Canada; ^4^ McMaster Institute for Research on Aging Hamilton ON Canada; ^5^ Department of Kinesiology University of Waterloo Waterloo ON Canada; ^6^ Schlegel‐UW Research Institute on Aging Waterloo ON Canada; ^7^ Department of Medicine McGill University Montreal QC Canada; ^8^ Department of Internal Medicine University of Manitoba Winnipeg MB Canada

**Keywords:** CLSA, COGNITION, FALLS, FRACTURES, FRAILTY

## Abstract

Cognition, frailty, and falls have been examined independently as potential correlates of fracture risk, but not simultaneously. Our objective was to explore the association between cognition, frailty, and falls and self‐reported incident fractures to determine if these factors show significant independent associations or interactions. We included participants who completed the Canadian Longitudinal Study on Aging (CLSA) 2012–2015 baseline comprehensive assessment, did not experience any self‐reported fractures in the year prior to cohort recruitment, and completed the follow‐up questionnaire at year 3 (*n* = 26,982). We compared all baseline cognitive measures available in the CLSA, the Rockwood Frailty Index (FI), and presence of self‐reported falls in the past 12 months in those with versus without self‐reported incident fractures in year 3 of follow‐up. We used multivariable logistic regression adjusted for covariates and examined two‐way interactions between cognition, frailty, and prior falls. CLSA specified analytic weights were applied. The mean ± standard error (SE) age of participants was 59.5 ± 0.1 years and 52.2% were female. A total of 715 participants (2.7%) self‐reported incident fractures at 3‐year follow‐up. Participants who experienced incident fractures had similar baseline cognition scores (mean ± SE; Rey Auditory Verbal Learning Test [RAVLT]: Immediate recall 6.1 ± 0.1 versus 5.9 ± 0.0; standardized difference [d] 0.124); higher FI scores (mean ± SE; FI 0.134 ± 0.005 versus 0.116 ± 0.001; d 0.193), and a greater percentage had fallen in the past 12 months (weighted *n* [%] 518 [7.2] versus 919 [3.5]; d 0.165). FI (each increment of 0.08) was associated with a significantly increased risk of self‐reported incident fractures in participants of all ages and those aged 65 years or older (adjusted odd ratio [OR] 1.24, 95% confidence limit [CL] 1.10–1.40; adjusted OR 1.44, 95% CL 1.11–1.52, respectively). The adjusted odds for self‐reported incident fractures in participants of all ages was also significantly associated with falls in the past 12 months prior to baseline (adjusted OR 1.83; 95% CL 1.13–2.97), but not in those aged 65 years or older. No interactions between cognition, frailty, and prior falls were found. However, considering the relatively young age of our cohort, it may be appropriate to make strong inferences in individuals older than 65 years of age. © 2022 The Authors. *JBMR Plus* published by Wiley Periodicals LLC on behalf of American Society for Bone and Mineral Research.

## Introduction

Osteoporosis is a disease characterized by compromised bone strength and an increased risk for fractures. Osteoporosis related fractures are associated with significant morbidity, mortality, and a high economic burden. After a hip fracture, 25% of people require institutionalization^(^ ^1^ ^)^ and over 30% will die.^(^ ^2^ ^)^ In Canada in 2014, the aggregate cost of osteoporosis attributable fractures was $4.6 billion.^(^ ^3^ ^)^ The estimated prevalence of osteoporosis in 2015–2016 for the Canadian population 40 years or older was 11.9% (approximately 2.2 million), and about 80% were women.^(^ ^4^ ^)^

Fracture risk factors, such as age, sex, bone mineral density, and prior fracture have been shown to be predictive of major osteoporotic fractures within the next 10 years.^(^ ^5^ ^)^ The Fracture Risk Assessment tool (FRAX)^(^ ^6^ ^)^ has been developed and tested to determine fracture risk for community‐dwelling older adults, to target appropriate health service planning and delivery. To date, FRAX has demonstrated satisfactory performance for predicting 10‐year fracture risk in Canadian men and women.^(^ ^7^ ^)^ In a clinical Canadian population, receiver operating characteristics curves for FRAX predicting hip and major osteoporotic fracture are 0.83 (95% confidence interval [CI] 0.82–0.85) and 0.69 (95% CI 0.68–0.71), respectively,^(^ ^7^ ^)^ demonstrating room for improvement in fracture risk prediction. Indeed, FRAX underestimates the risk of fracture for people with a history of falls and there have been suggestions to add falls as a risk factor to FRAX.^(^ ^8^ ^)^ Our previous work has demonstrated that cognition, falls, and elements of frailty are important risk factors for fractures for older adults living in long‐term care homes.^(^ ^9^ ^)^ FRAX currently does not consider cognition and frailty, and it is unknown whether including them in fracture risk assessment would improve the 10‐year probability estimate of hip and major osteoporotic fracture in adults residing in a community setting.

Cognitive impairment has been associated with an increased risk of fracture. Women with mild cognitive impairment have been found to have a 1.59‐fold higher risk of hip fracture compared to women without cognitive impairment when adjusting for fracture site, age, and race.^(^ ^10^ ^)^ Cognitive decline has also been demonstrated to be an independent risk factor for falls.^(^ ^11^, ^12^ ^)^ For example, older adults with impaired executive function were three times more likely to fall during a 2‐year follow‐up.^(^ ^12^ ^)^ It is unclear whether there is an independent association between cognition and fractures, or if this association is because people with cognitive impairment have an increased risk for falls. Additionally, cognition is often included in frailty indices (FIs),^(^ ^13^ ^)^ making it challenging to determine if cognitive impairment alone is associated with fracture. Frailty is defined as a state of diminished reserves (ie, physical, health, cognition) that results in vulnerability and adverse outcomes.^(^ ^14^, ^15^ ^)^ Frailty has been shown to be an independent predictor of hip fractures.^(^ ^16^, ^17^, ^18^, ^19^ ^)^ Frailty can also increase the risk of future cognitive impairment^(^ ^20^ ^)^ and is a predictor of falls.^(^ ^21^ ^)^ Like frailty, self‐reported falls have been demonstrated to be an independent risk factor for fracture in women (hazard ratio [HR] 1.64, 95% CI 1.20–2.24)^(^ ^22^ ^)^ and men (HR 3.47, 95% CI 1.02–11.80).^(^ ^23^ ^)^

Cognition, frailty, and falls have been examined independently in relation to fracture risk and to each other but have not been considered simultaneously in models using population‐based data. It is important to examine the variables simultaneously because it may be that the interactions between them are important for fracture prediction. Therefore, our objective was to examine the association between cognition, frailty, and falls and self‐reported incident fractures in year 3 of follow‐up to determine if these factors show independent associations or significant interactions.

## Subjects and Methods

### The Canadian Longitudinal Study on Aging

The Canadian Longitudinal Study on Aging (CLSA) evaluates factors affecting the aging process to improve the health of older Canadian. The CLSA is a national, stratified, random sample of 51,338 (including 30,097 from “comprehensive” cohort and 21,241 from “tracking” cohort) community‐dwelling men and women aged 45 to 85 years across the 10 Canadian provinces.^(^ ^24^ ^)^ All CLSA participants provide extensive questionnaire data and a subset, the “comprehensive” cohort (*n* = 30,097) and also undergo in‐depth physical assessments at one of 11 data collection sites (DCSs) across Canada: Vancouver/Surrey, Victoria, Calgary, Winnipeg, Hamilton, Ottawa, Montreal, Sherbrooke, Halifax, St. John's.^(^ ^24^ ^)^ The CLSA has collected biological, medical, psychological, social, lifestyle, and economic data every 3 years for 20 years from 2010. Excluded from the CLSA sampling frame were individuals residing in the three Canadian territories, individuals living on federal First Nations reserves, full‐time members of the Canadian Armed Forces, individuals unable to provide data in English or French, individuals unable to provide informed consent and/or data from themselves (eg, with cognitive impairment), and individuals living in institutions (eg, long‐term care facilities). Our study included only a cohort of 30,097 that had data collection and home interview, versus the other 21,241 that included telephone only.

### Participants with self‐reported new fractures in the last year at follow‐up

**Fig. 1:**
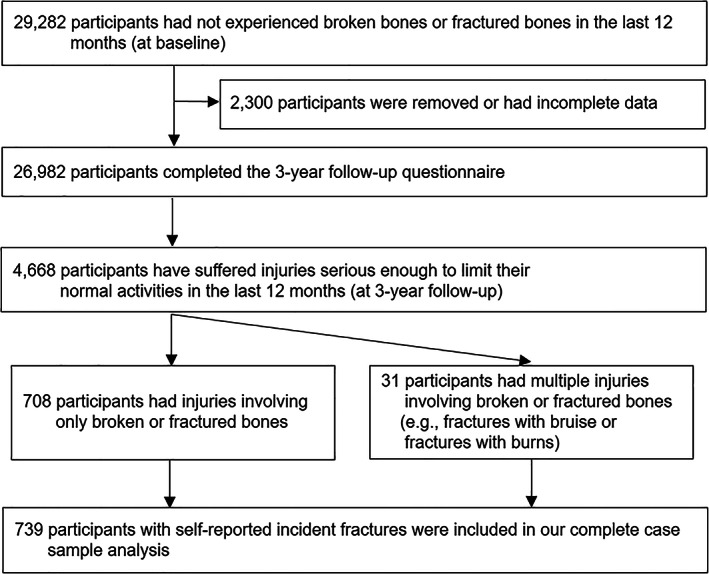
Selection process of participants in this study.

We included participants from the comprehensive cohort who did not experience any self‐reported fractures in the year prior to their baseline interview and completed the first follow‐up (*n* = 26,982) (Fig. 1).

Of the 29,282 participants who had not experienced any fractures in the past year at baseline, 26,982 followed for 3 years were included. Among them, 4668 participants experienced injuries that may limit normal activities over the past year of which 739 participants self‐reported incident fractures, including those who experienced fractures (*n* = 708) and those who experienced fractures as part of multiple injuries (*n* = 31) (eg, fractures with bruises, fractures with burns, etc.) (Fig. 1).

We included all types of fractures rather than only osteoporotic fractures. When asked which skeletal sites were fractured, almost all (ie, about 96%) did not respond. Therefore, we could not classify fractures in consideration of skeletal sites.

### Cognition

We included all domains of the cognitive measures in the CLSA: (i) memory (ie, Rey Auditory Verbal Learning Test [RAVLT]: immediate recall and 5‐minute delayed recall),^(^ ^25^ ^)^ (ii) executive function (ie, Mental Alteration Test [MAT],^(^ ^26^ ^)^ Animal fluency: strict,^(^ ^27^ ^)^ Controlled Oral Word Association (COWAT),^(^ ^28^ ^)^ Stroop test: Victoria version,^(^ ^29^, ^30^ ^)^ and Miami Prospective Memory Test [MPMT]),^(^ ^31^ ^)^ and (iii) psychomotor speed (ie, Choice Reaction Time Ask [CRT]).^(^ ^32^ ^)^ Three cognitive measures (ie, RAVLT, MAT, and Animal fluency) were conducted during a 90‐minute in‐home interview whereas the others (ie, COWAT, Stroop test, MPMT, and CRT) were administered at the DCS visit.^(^ ^33^ ^)^ The three domains were included because each domain is associated with adaptive functioning during one's lifespan, a gradual age‐related normative decline has been shown for each domain, significant changes in each domain has been related to age‐related medical conditions, and each domain may be related to specific genetic markers.^(^ ^34^ ^)^ Executive function, especially, engages in many complex behaviors, including mental flexibility, ability to respond, and response inhibition, and is involved in any task that allows people to engage in independent adaptive behavior.^(^ ^34^ ^)^

Table 1 shows the range of scores, measurement methods and characteristics of each cognitive measure in this analysis. Cognition variables are generally recommended to be converted from raw scores on the various test measures to demographically corrected *T*‐scores because of high correlation with neuropsychological test performance (eg, sex, age, and years of education).^(^ ^35^ ^)^ However, we did not construct normative standards for cognitive variables because we believed that also including sex, age, and years of education as covariates might lead to overadjustment in the regression model. In addition, even though two types (ie, strict and lenient) of Animal fluency scores are available in the CLSA, only the strict Animal fluency was included in this study because of multicollinearity when testing with other cognitive measures.

**Table 1:** Summary of Scores, Measurement Methods and Characteristics for the Cognitive Measures in this Study

Cognitive measures	Range of score	Measurement methods and characteristics
Memory		
RAVLT: immediate recall^(^ ^25^ ^)^	0–15	The RAVLT is a 15‐items word learning test for assessment of learning and retention, where participants were tested for immediate memory recall and delayed memory recall approximately 5 minutes later.^(^ ^33^, ^52^ ^)^ Higher values reflect better performance.
RAVLT: 5‐min delayed recall^(^ ^25^ ^)^	0–15	
Executive function		
Animal Fluency Test: strict^(^ ^27^ ^)^	0–52	For Animal fluency test, participants were asked to name as many animals (based on animals' scientific taxonomic classification) as possible in 60 seconds.^(^ ^33^, ^52^ ^)^ Higher values reflect better performance.
MAT^(^ ^26^ ^)^	0–52	The MAT, which is known as a brief cognitive switching task, assesses mental flexibility and processing speed through a score that sums number of corrected alternations of matched pairs of the alphabet and numbers (eg, 1‐A, 2‐B) within 30 seconds.^(^ ^33^, ^52^ ^)^ Higher values reflect better performance.
MPMT: total^a^ ^(^ ^31^ ^)^	0–18	For MPMT, participants were asked to complete each task (ie, event‐based, which is completed in the presence of an external cue, and timed‐based, which is carried out after a set amount of time, or at a specific time) at the sound of each task which was combined with the scores of two tasks.^(^ ^33^, ^52^ ^)^ For event‐based task, an examiner showed a participant an envelope containing bills and change, and when the timer rang, the participant was asked to give to the examiner a five‐dollar bill and to keep the ten‐dollar bill in the envelope. For timed‐based task, fifteen minutes after start of the MPMT (ie, 8:15), the examiner asked the participant to give the card number 17 from the five numbered cards (ie, 28, 14, 17, 13, 11).^(^ ^55^ ^)^ Higher values reflect better performance.
COWAT: total^b^ ^(^ ^28^ ^)^	3–105	For COWAT, participants were asked to name as many words as they could that begin with “F”, “A” and “S” within 60 seconds.^(^ ^33^, ^52^ ^)^ Higher values reflect better performance.
Stroop Test: inference ratio (color/dot)^c^ ^(^ ^29^, ^30^ ^)^	0.05–38.06	For the Stroop test, participants were asked to respond to the color of ink on stimulus cards (ie, colored dots, common words printed in same colors as dots, and color words printed in non‐corresponding colors of ink).^(^ ^33^, ^52^ ^)^ An interference ratio which divided the time required to complete the last card (ie, color) by the time required to complete the first card (ie, dot).^(^ ^33^, ^52^ ^)^ Lower values reflect better performance.
Psychomotor speed		For CRT, participants were asked to press a key on a touch screen as quickly and accurately as possible.^(^ ^33^, ^52^ ^)^ The scores of the correct answers excluding incorrect answers and timeouts were used.^(^ ^33^, ^52^ ^)^ Lower values reflect better performance.
CRT: mean response time (ms)^(^ ^32^ ^)^	79–9958	

COWAT = Controlled Oral Word Association Test; CRT = Choice Reaction Time; MAT = Mental Alternation Test; MPMT = Miami Prospective Memory Test; RAVLT = Rey Auditory Verbal Learning Test.

^a^ MPMT – Total is sum of 30‐min event‐based (scores 0–9) and 15‐min timed‐based score (scores 0–9), which each based included intention to perform (scores 0–3), accuracy of response (scores 0–3), and need for reminders (scores 0–3).

^b^ COWAT – Total is sum of total number of acceptable words beginning with F (scores 0–35 for English; scores 0–31 for French), A (scores 0–35 for English; scores 0–30 for French), and S (scores 0–40 for English; scores 0–29 for French) in 60 seconds.

^c^ Stroop test: inference ratio (color/dot) is total number of seconds to complete color (seconds 0–609 for English; seconds 0–224 for French) divided by total number of seconds to complete dot (seconds 0–111 for English; seconds 0–48 for French).

### Frailty

The Rockwood FI was calculated as the proportion of age‐related health deficits present in an individual out of the total potential deficits assessed.^(^ ^15^, ^36^ ^)^ The FI was constructed based on standard procedures previously used in the CLSA.^(^ ^36^ ^)^ The FI consisted of 42 variables at baseline: activities of daily living (*n* = 9), depression (*n* = 1), physical activity (*n* = 5), perceived health (*n* = 1), chronic disease (*n* = 23), social participation (*n* = 1), and vision/hearing (*n* = 2). A total of 28 items that inversely correlated with age (eg, allergies, asthma, epilepsy, migraine headaches or overactive thyroid gland) or correlated with cognition (eg, dementia, Alzheimer disease, or memory problems) were excluded. In accordance with Searle and colleagues,^(^ ^36^ ^)^ we included only variables showing that a deficit's prevalence increases with age. In addition, we tested plotting the mean of the items against age through the Spearman correlation coefficients.^(^ ^37^ ^)^ The FI was calculated by adding up the values of deficits and dividing by the total number of items (*n* = 42), with the FI ranging from 0 to 1. For example, if an individual had eight deficits of 42 considered, the FI score would be 8/42 = 0.19. FI was calculated only if there were less than two missing items out of 42 items (ie, about 5%), and if there were more than two missing items, it was dealt with as missing values. None of the included deficit variables had more than 5% missing values.

### Falls

Participants were asked “How many times have you fallen in the last 12 months?” Based on this, participants were classified as a non‐faller (no) if they had not experienced a fall, and faller (yes) if they had at experienced at least one fall.

### Statistical analysis

According to CLSA recommendation,^(^ ^38^ ^)^ we used the analytic weights in order to decrease the effect of selection bias introduced during the sampling process on the estimates and to improve generalizability of the results. We used weighted mean (standard errors [SE]) and weighted frequency (percentages) to describe continuous and categorical data, respectively.

Because the results of the difference test of population parameters may vary largely depending on the sample size,^(^ ^39^ ^)^ we tested standardized difference, a unified approach that quantifies the magnitude of differences between groups of baseline variables.^(^ ^39^ ^)^ According to Cohen,^(^ ^40^ ^)^ we interpreted the effect size indices of 0.2, 0.5, and 0.8, respectively, as small, moderate, and large effects.

We analyzed the association between each of the cognitive measures, FI, and a history of falling at least once or not falling, and incident self‐reported fractures reported at follow‐up (approximately 3 years later) using multivariable logistic regression model. We calculated odds ratio (OR) and 95% confidence limit (CL). We reported the ORs measured per‐0.08 (standard deviation [SD] = 0.08, approximately 1 SD) increment in the FI which is consistent with other studies.^(^ ^13^ ^)^ We also reported the ORs measured per‐one unit change (ie, 1 SD) in each cognitive measure. We conducted a subgroup analysis for participants aged 65 years or older. We did not include variables in the analysis if the Variance Inflation Factor (VIF) was 10 or higher indicating significant multicollinearity. Two‐way interaction terms within each independent variables (ie, cognitive measures * FI, FI * history of falls, and history of falls * cognitive measures), between the independent variables and age (ie, cognitive measures * age, FI * age, and history of falls * age) and between the independent variables and sex (ie, cognitive measures * sex, FI * sex, and history of falls * sex) were tested. We adjusted our models for the following traditional fracture risk factors: age group (45–54, 55–64, 65–74, ≥75 years), sex (male, female), body mass index (BMI) group (underweight:<18.5 kg/m^2^, normal weight:18.5–24.9 kg/m^2^, overweight: 25.0–29.9 kg/m^2^, obese: ≥30.0 kg/m^2^), ethnicity (white, non‐white), education level (less than secondary school graduation, secondary school graduation, or no postsecondary education), household income in past 12 months (<$20,000, $20,000–49,999, $50,000–$99,999, $100,000–$149,999, ≥$150,000), marital status (single or never married, married, widowed or divorced or separated), smoking status (nonsmoker, past smoker, current smoker), alcohol consumption in past 12 months (never, less than once a month, 1–4 times a month, 2–5 times a week, almost every day), parental hip fracture history (yes, no), any previous fractures during adult life (yes, no), corticosteroid use (yes, no), self‐reported rheumatoid arthritis (yes, no), self‐reported osteoporosis (yes, no), and dual‐energy X‐ray absorptiometry (DXA) femoral neck *T*‐score.

We conducted multiple imputation with 10 replications^(^ ^41^ ^)^ to handle missing data (eg, 7.4% of DXA femoral neck *T*‐score and 6.2% of household income data were imputed). We also conducted sensitivity analysis to compare complete case analysis for non‐weighted case and weighted case (Table S2).

We used two‐sided tests with a significance level of 0.05. All analyses were conducted in SAS v9.4 (SAS Institute, Cary, NC, USA).

## Results

Of the total 26,754 participants at 3‐year follow‐up, 715 had self‐reported incident fractures. Participants with self‐reported incident fractures were older (mean ± SE, 60.4 ± 0.6 years versus 59.5 ± 0.1 years) were more often female (63.5% versus 51.9%), had higher scores on the RAVLT: immediate recall (mean ± SE, 6.1 ± 0.1 versus 5.9 ± 0.0), had higher mean FI (mean ± SE, 0.134 ± 0.005 versus 0.116 ± 0.001) and more experienced a fall in the past 12 months (7.2% versus 3.5%) compared to those without (Table 2). Participants' baseline characteristics and comparisons between participants with and without self‐reported new fractures in the last year at follow‐up for non‐weighted case were shown in Table S1.

**Table 2:** Participants’ Baseline Characteristics and Comparisons Between Participants With and Without Self‐Reported Incident Fractures in the Last Year of Follow‐Up (Weighted Case)

		Participants with self‐reported new fractures		
Characteristics	Participants of all ages (*n* = 26,754)^a^	Yes (*n* = 715)^b^	No (*n* = 26,039)^c^	Standardized difference^d^
Age (years), mean ± SE	59.5 ± 0.1	60.4 ± 0.6	59.5 ± 0.1	0.081
Age (years) group, *n* (%)				0.146
45 to 54	10,506 (39.3)	270 (37.7)	10,237 (39.3)	
55 to 64	8560 (32.0)	229 (32.0)	8332 (32.0)	
65 to 74	4879 (18.2)	108 (15.1)	4771 (18.3)	
≥75	2808 (10.5)	109 (15.2)	2699 (10.4)	
Female sex, *n* (%)	13,973 (52.2)	454 (63.5)	13,519 (51.9)	−0.235
BMI (kg/m^2^), mean ± SE	28.4 ± 0.1	27.87 ± 0.3	28.45 ± 0.1	−0.122
BMI (kg/m^2^) group, *n* (%)				−0.134
Underweight (<18.5 kg/m^2^)	180 (0.7)	3 (0.4)	177 (0.7)	
Normal weight (18.5–24.9 kg/m^2)^	7509 (28.2)	235 (32.8)	7274 (28.0)	
Overweight (25.0–29.9 kg/m^2^)	10,396 (39.0)	291 (40.7)	10,106 (39.0)	
Obese (≥30.0 kg/m^2^)	8562 (32.1)	187 (26.1)	8375 (32.3)	
Ethnicity, *n* (%)				−0.066
White	23,653 (84.7)	589 (82.3)	22,064 (84.7)	
Non‐white	4101 (15.3)	127 (17.7)	3975 (15.3)	
Education, *n* (%)				−0.024
Less than secondary school graduation	4120 (15.4)	98 (13.8)	4022 (15.5)	
Secondary school graduation, no postsecondary education	3021 (11.3)	81 (11.4)	2940 (11.3)	
Some postsecondary education	2494 (9.3)	85 (11.9)	2409 (9.3)	
Postsecondary degree/diploma	17,087 (63.9)	449 (62.9)	16,638 (64.0)	
Household income in past 12 months, *n* (%)				0.007
<$20,000	1416 (5.6)	41 (6.2)	1374 (5.6)	
$20,000 to $49,999	5436 (21.6)	179 (27.1)	5256 (21.4)	
$50,000 to $99,999	8721 (34.6)	217 (32.7)	8504 (34.6)	
$100,000 to $149,999	5189 (20.6)	104 (15.7)	5084 (20.7)	
≥$150,000	4.449 (17.6)	121 (18.2)	4328 (17.6)	
Marital status, *n* (%)				0.131
Single, never married	1838 (7.1)	43 (6.1)	1795 (7.1)	
Married	19,558 (75.3)	495 (71.1)	19,063 (75.5)	
Widowed, divorced, separated	4565 (17.6)	158 (22.7)	4407 (17.4)	
Smoking status, *n* (%)				0.076
Nonsmoker	11,938 (44.6)	313 (43.7)	11,625 (44.6)	
Past smoker	11,808 (44.1)	305 (42.6)	11,503 (44.2)	
Current smoker	3008 (11.2)	98 (13.7)	2910 (11.2)	
Alcohol consumption in past 12 months, *n* (%)				0.104
Never	3187 (12.2)	67 (9.6)	3120 (12.3)	
Less than once a month	3560 (13.6)	96 (13.8)	3464 (13.6)	
1 to 4 times a month	7560 (29.0)	192 (27.7)	7368 (29.0)	
2 to 5 times a week	8068 (30.9)	215 (30.9)	7853 (30.9)	
Almost every day	3723 (14.3)	126 (18.1)	3597 (14.2)	
Parental hip fracture history, *n* (%)	3110 (11.8)	94 (13.4)	3016 (11.8)	0.048
Previous fractures during adult life, *n* (%)	3422 (12.8)	176 (24.6)	3246 (12.5)	0.315
Corticosteroids use, *n* (%)	3375 (12.9)	122 (17.2)	3253 (12.7)	0.125
Self‐reported rheumatoid arthritis status, *n* (%)	872 (3.3)	52 (7.4)	820 (3.2)	0.189
Self‐reported osteoporosis status, *n* (%)	1980 (7.5)	104 (14.7)	1876 (7.3)	0.240
DXA femoral neck *T*‐score, mean ± SE	−0.57 ± 0.01	−0.97 ± 0.06	−0.56 ± 0.01	−0.351
Osteoporosis category based on WHO classification, *n* (%)				0.176
Normal (*T*‐score ≥ −1)	15,829 (59.5)	334 (46.8)	15,595 (59.9)	
Osteopenia (−2.5 < *T*‐score < −1)	8162 (30.5)	276 (38.6)	7886 (30.3)	
Osteoporosis (*T*‐score ≤ −2.5)	3150 (10.0)	105 (14.6)	2558 (9.8)	
Cognitive measures, mean ± SE				
RAVIT: immediate recall	5.9 ± 0.02	6.1 ± 0.09	5.9 ± 0.02	0.124
RAVIT: 5‐minute delayed recall	4.1 ± 0.02	4.3 ± 0.12	4.1 ± 0.02	0.111
Animal fluency test: strict	19.7 ± 0.05	19.9 ± 0.29	19.7 ± 0.05	0.001
MAT	26.5 ± 0.08	26.5 ± 0.44	26.5 ± 0.08	−0.002
MPMT: total	17.1 ± 0.02	17.2 ± 0.08	17.1 ± 0.02	0.008
Stroop test: interference ratio (color/dot)^e^	2.1 ± 0.01	2.2 ± 0.03	2.1 ± 0.01	−0.051
COWAT: total	40.0 ± 0.09	38.96 ± 0.72	39.97 ± 0.11	0.066
CRT: mean response time (ms)^e^	827.1 ± 1.94	831.01 ± 9.46	827.01 ± 1.98	−0.056
Frailty index, mean ± SE	0.116 ± 0.001	0.134 ± 0.005	0.116 ± 0.001	0.193
History of falls, *n* (%)				0.165
Non‐faller (no)	25,783 (96.4)	663 (92.8)	25,118 (96.5)	
Faller (yes)	971 (3.6)	52 (7.2)	919 (3.5)	

Animal fluency test (range, 0–52). BMI = body mass index; COWAT = Controlled Oral Word Association Test (range, 3–105); CRT = Choice Reaction Time (range, 79–9958); MAT = Mental Alternation Test (range, 0–52); MPMT = Miami Prospective Memory Test (range, 0–18); RAVLT = Rey Auditory Verbal Learning Test (range, 0–15).

^a^ A total of 26,982 participants were included, which are non‐weighted numbers.

^b^ 739 participants with self‐reported fractures were included, which are non‐weighted numbers.

^c^ 26,243 participants without self‐reported fractures were included, which are non‐weighted numbers.

^d^ Standardized difference is difference in means or proportions divided by standard error. Imbalance defined as absolute value greater than 0.20 (small effect size).

^e^ Lower values reflect better performance.

Table 3 shows the results of the adjusted multivariable logistic regression for the relationship between baseline cognitive measures, FI, a history of falls and self‐reported incident fractures following multiple imputation. For each SD increment in FI (SD = 0.08, approximately 1 SD) the adjusted odds for self‐reported incident fractures was 1.24 (95% CL 1.10–1.40, *p* value <0.001) in participants of all ages. The adjusted odds for self‐reported incident fractures in participants of all ages was significantly associated with history of falls at baseline 1.83 (95% CL 1.13–2.97, *p* value 0.014). RAVLT: immediate recall of the cognitive measures was associated with self‐reported incident fractures in participants of all ages after adjusting for covariates (adjusted OR 1.09 per 1.89 increment, 95% CL 1.01–1.18, *p* value 0.033), but other cognitive measures were not associated. For participants aged 65 years or older, FI was significantly associated with self‐reported incident fractures (adjusted OR 1.44 per 0.08 increment, 95% CL 1.11–1.52, *p* value 0.001), but history of falls was not (adjusted OR 1.44, 95% CL 0.76–2.73, *p* value 0.258). Likewise, MPMT was associated with self‐reported incident fractures in those aged 65 years or older (adjusted OR 1.07 per 2.41 increment, 95% CL 1.00–1.14, *p* value 0.038), but other cognitive measures were not (Table 3). In addition, there were no significant interactions within three factors (ie, cognitive measures, FI, and history of falls), between them and age, and between them and sex in both participants of all ages and those aged 65 years or older (Table 4).

**Table 3:** Adjusted ORs With 95% CLs for the Relationship Between Cognition, Frailty, Falls, and Self‐Reported Incident Fractures: Multiple Imputation Analysis (Weighted Case)

Variables	Adjusted OR^a^ (95% CLs)	*p*
Participants of all ages (*n* = 26,754)		
Cognitive measures		
RAVLT: immediate recall (per‐1.89 [one SD] increment)	1.09 (1.01–1.18)	0.033
RAVLT: 5‐minute delayed recall (per‐2.16 [one SD] increment)	0.98 (0.90–1.05)	0.503
Animal fluency test: strict (per‐5.64 [one SD] increment)	1.01 (0.99–1.03)	0.490
MAT (per‐8.65 [one SD] increment)	1.00 (0.99–1.02)	0.827
MPMT (per‐1.98 [one SD] increment)	1.05 (0.99–1.10)	0.082
COWAT (per‐12.84 [one SD] increment)	1.00 (0.99–1.01)	0.593
Stroop test: interference ratio (color/dot) (per‐0.71 [one SD] increment)	1.00 (0.89–1.11)	0.938
CRT: mean response time (per‐238.52 [one SD] increment)	1.00 (1.00–1.00)	0.469
Frailty index (per‐0.08 [one SD] increment)	1.24 (1.10–1.40)	<0.001
History of falls		
Non‐faller (no)	Reference	
Faller (yes)	1.83 (1.13–2.97)	0.014
Participants aged over 65 years (*n* = 7687)		
Cognitive measures		
RAVLT: immediate recall (per‐1.79 [one SD] increment)	1.10 (0.99–1.23)	0.078
RAVLT: 5‐minute delayed recall (per‐1.98 [one SD] increment)	0.95 (0.87–1.05)	0.325
Animal fluency test: strict (per‐5.15 [one SD] increment)	1.01 (0.98–1.04)	0.669
MAT (per‐8.53 [one SD] increment)	1.00 (0.98–1.03)	0.687
MPMT (per‐2.41 [one SD] increment)	1.07 (1.00–1.14)	0.038
COWAT (per‐12.89 [one SD] increment)	1.00 (0.99–1.02)	0.660
Stroop test: interference ratio (color/dot) (per‐0.77 [one SD] increment)	1.03 (0.83–1.27)	0.819
CRT: mean response time (per‐271.94 [one SD] increment)	1.00 (1.00–1.00)	0.268
Frailty index (per‐0.08 [one SD] increment)	1.44 (1.11–1.52)	0.001
History of falls		
Non‐faller (no)	Reference	
Faller (yes)	1.44 (0.76–2.73)	0.258

CL = confidence limit; COWAT = Controlled Oral Word Association Test; CRT = Choice Reaction Time; MAT = Mental Alternation Test; MPMT = Miami Prospective Memory Test; RAVLT = Rey Auditory Verbal Learning Test.

^a^ Adjusted for age, sex, ethnicity, education level, marital status, income, smoking, alcohol consumption, BMI group, parental hip fracture history, prior fracture, corticosteroids use, self‐reported rheumatoid arthritis status, self‐reported osteoporosis status and DXA femoral neck *T*‐score.

**Table 4:** Results for Interactions Within Independent Variables and Between Independent Variables, Age, or Sex

	Participants of all ages (*n* = 26,754) *p*	Those aged over 65 years (*n* = 7687) *p*
Interaction terms within independent variables		
RAVLT: immediate recall × frailty index	0.668	0.722
RAVLT: 5‐minute delayed recall × frailty index	0.210	0.211
Animal fluency test: strict × frailty index	0.091	0.891
MAT × frailty index	0.096	0.940
MPMT: total × frailty index	0.616	0.130
COWAT: total × frailty index	0.894	0.607
Stroop test: interference ratio (color/dot) × frailty index	0.497	0.415
CRT: mean response time × frailty index	0.851	0.494
History of falls × frailty index	0.893	0.972
RAVLT: immediate recall × history of falls	0.949	0.213
RAVLT: 5‐minute delayed recall × history of falls	0.655	0.898
Animal fluency test: strict × history of falls	0.300	0.325
MAT × history of falls	0.125	0.561
MPMT: total × history of falls	0.768	0.870
COWAT: total × history of falls	0.501	0.147
Stroop test: interference ratio (color/dot) × history of falls	0.361	0.531
CRT: mean response time × history of falls	0.574	0.318
Interaction terms between independent variables and age		
RAVLT: immediate recall × age	0.350	0.757
RAVLT: 5‐minute delayed recall × age	0.230	0.305
Animal fluency test: strict × age	0.364	0.105
MAT × age	0.299	0.933
MPMT: total × age	0.811	0.277
COWAT: total × age	0.847	0.301
Stroop test: interference ratio (color/dot) × age	0.541	0.693
CRT: mean response time × age	0.316	0.409
Frailty index × age	0.411	0.184
History of falls × age	0.161	0.324
Interaction terms between independent variables and sex		
RAVLT: immediate recall × sex	0.350	0.994
RAVLT: 5‐minute delayed recall × sex	0.537	0.123
Animal fluency test: strict × sex	0.621	0.942
MAT × sex	0.995	0.923
MPMT: total × sex	0.450	0.707
COWAT: total × sex	0.917	0.637
Stroop test: interference ratio (color/dot) × sex	0.105	0.691
CRT: mean response time × sex	0.873	.0408
Frailty index × sex	0.914	0.889
History of falls × sex	0.251	0.613

COWAT = Controlled Oral Word Association Test; CRT = Choice Reaction Time; MAT = Mental Alternation Test; MPMT = Miami Prospective Memory Test; RAVLT = Rey Auditory Verbal Learning Test.

Similar to our main results, the sensitivity analysis showed that the FI was significantly associated with self‐reported incident fractures, with an adjusted OR of 1.21 (95% CI 1.09–1.34, *p* value <0.001) and 1.25 (95% CL 1.07–1.48, *p* value 0.006) for an increase of per‐0.08 (approximately 1 SD) in complete case analysis with both non‐weighed and weighted case (Table S2). In contrast with our main results, the sensitivity showed that history of falls was not associated with self‐reported incident fractures in complete case analysis with weighted case with an adjusted OR of 1.81 (95% CL 0.99–3.31, *p* value 0.053), but the adjusted OR was similar with adjusted OR in multiple imputation analysis (Table S2). In addition, none of the cognitive measures were significantly associated with self‐reported incident fractures after adjusting for the covariates in the sensitivity analysis (Table S2). The results of the sensitivity analysis in participants aged 65 years or older were similar to those of participants of all ages. FI was significantly associated with self‐reported incident fractures in complete case analysis with both non‐weighted and weighted case (adjusted OR 1.31, 95% CI 1.10–1.55, *p* value 0.002; adjusted OR 1.40, 95% CL 1.12–1.74, *p* value 0.003, respectively) (Table S3). However, in participants aged 65 years or older, the association between history of falls and self‐reported incident fractures was significant in complete case analysis with non‐weighted case (adjusted OR 2.40, 95% CI 1.33–4.31, *p* value 0.004), but not with weighted case (adjusted OR 1.87, 95% CL 0.95–3.69, *p* value 0.071) (Table S3). No cognitive measures were significantly associated with self‐reported incident fractures, and no interactions were found to be significant. Multicollinearity of independent variables was not shown (Tables S4 and S5).

## Discussion

FI and history of falls were independently associated with fractures in the primary analyses. In addition, RAVLT: immediate recall of the cognitive measures was associated with fractures. There were no significant interactions between cognitive measures, FI, and history of falls. Since our cohort was relatively young, we explored the association between cognition, frailty, falls, and self‐reported incident fractures in participants aged 65 years or older as subgroup analysis. FI was significantly associated with self‐reported incident fractures, but history of falls was not. In addition, only MPMT was significantly associated with self‐reported incident fractures for participants aged 65 years or older.

Our results demonstrate that frailty was independently associated with self‐reported incident fractures in both participants of all ages and those aged 65 years or older. Other studies have demonstrated frailty has been associated with fractures. The Global Longitudinal Study of Osteoporosis in Women (GLOW) 3‐year Hamilton cohort showed that the HR for major osteoporotic fracture per‐0.01 increment for FI was 1.02 (95% CI 1.01–1.04).^(^ ^42^ ^)^ The Canadian Multicentre Osteoporosis Study (CaMos) demonstrated over the 10‐year study period the HR was 1.25 (*p* value <0.001) for all fractures, 1.18 (*p* value 0.043) for hip fractures, and 1.30 (*p* value 0.001) for clinical vertebral fractures per‐0.10 increment for FI.^(^ ^13^ ^)^ Our result was also consistent in other studies that used frailty phenotype. A multiple country study from the GLOW^(^ ^17^ ^)^ showed that adjusted OR for frailty phenotype (frail versus non‐frail) was 1.23 (95% CI 1.07–1.42) for fracture. In addition, a longitudinal cohort study of Women's Health Initiative (WHI) showed that frailty phenotype was associated with hip fracture (HR 1.87, 95% CI 1.02–3.42).^(^ ^19^ ^)^ On the other hand, other population based studies on aging have not demonstrated an association between frailty and fractures. The Beijing Longitudinal Study on Aging (BLSA)^(^ ^43^ ^)^ showed that the FI was not associated with an increased risk of recurrent fractures (OR 1.07 per each increment, 95% CI 0.94–1.22), which was because FI of the oldest age group was the highest whereas the proportion of people with fractures was the lowest in the group. The Longitudinal Aging Study Amsterdam (LASA)^(^ ^44^ ^)^ showed that frailty measured by nine markers (eg, BMI and depressive symptoms), which was similar with the frailty phenotype, was not associated with more than one fracture (adjusted OR 1.22, 95% CI 0.84–1.79) due to a small number of fractures during follow‐up.

Our findings confirm that a history of falls was associated with self‐reported incident fractures in participants of all ages, which is also consistent with previous studies.^(^ ^22^, ^45^, ^46^ ^)^ The Hertfordshire cohort study in the UK^(^ ^45^ ^)^ showed that a history of falls was related to fractures for men (adjusted HR 6.75, 95% CI 3.41–13.36) and women (adjusted HR 6.33, 95% CI 3.50–11.44). The Nord‐Trondelag Health Study (HUNT) in Norway^(^ ^22^ ^)^ showed an association only for women aged 50–90 years (adjusted HR 1.64, 95% CI 1.20–2.24). Sex is an important covariate, as the previous studies have shown that the relationship between a history of falls and fractures varies slightly by sex. Thus, our study adjusted for sex in our multivariable logistic model and showed a significant relationship. A meta‐analysis of the Osteoporotic Fractures in Men (MrOS) international cohort study from Sweden, Hong Kong, and the United States^(^ ^46^ ^)^ also reported that a history of falls predicted any incident fractures (adjusted HR 1.69, 95% CI 1.49–1.90). In addition to the results of the previous studies, our results may support a report from the International Society of Clinical Densitometry/International Osteoporosis Foundation Task Force which suggested that modification of FRAX probability by considering a history of falls may improve the 10‐year hip fracture risk by 30% (multiplied by 1.3) for each previous fall (for up to 5 falls).^(^ ^8^ ^)^ Our study did not find that a history of falls was associated with self‐reported incident fractures in those aged 65 years or older. This may be because the proportion of self‐reported incident fractures in those aged 65 years or older with a history of falls (weighted *n* = 13; 6%) was slightly less than that for all aged participants (weighted *n* = 52; 7.2%).

In terms of cognition, some previous studies show similar results to ours. The relationship between cognition and incident fracture is not as clear in our study or in the literature. Our results suggest that participants who have higher cognitive performance (ie, RAVLT: Immediate recall in participants of all ages and MPMT in those aged 65 years or older) may be more likely to experience a fracture. It maybe because participants who are less cognitively impairment are more independently mobile and have more opportunities to fall and fracture. In addition, given that all other cognitive measures evaluated in the current study were not associated with fractures, these results should be interpreted carefully and additional research is need to determine the association between cognition and fracture. The Study of Osteoporotic Fractures (SOF) Research Group^(^ ^10^ ^)^ reported that women with mild cognitive impairment measured from comprehensive neuropsychological tests including Mini‐Mental State Examination (MMSE) had a 1.48‐fold higher risk of hip fracture (adjusted HR 1.48, 95% CI 0.98–2.28) but this was not statistically significant. Another 10‐year follow‐up prospective cohort study^(^ ^47^ ^)^ demonstrated that for women aged 75 years or older poor cognitive status (MMSE ≤ 23) was significantly associated with fractures (relative ratio [RR] 1.80, 95% CI 1.30–2.40), while for women aged 65 to 74 years this was not significant (RR 0.90, 95% CI 0.70–1.20). Most prospective studies^(^ ^48^, ^49^, ^50^, ^51^ ^)^ evaluating the association between cognition and fracture risk did not include rigorous evaluation of cognitive status^(^ ^10^ ^)^ or used different tools when evaluating cognition, making it difficult to compare with our findings. Further, participants in the CLSA tended to be socially advantaged and healthier because those with low levels of literacy in English or French (including recent immigrants), or with hearing or memory problems were less likely to participate or score well on cognitive measures.^(^ ^24^ ^)^ Additionally, a study^(^ ^52^ ^)^ explaining the implementation of cognitive measures in CLSA showed that the mean values of each cognitive measure in CLSA were similar or slightly higher in CLSA compared to those of healthy older adults in other previous studies. Finally, dementia is related to age with impaired cognition usually increasing with age, but the majority (ie, 71.3%) of the participants in our study were under 65 years old. Given the young average age (ie, 59.5 years old) of the participants, cognition may not have been associated with fractures since our data lacked variability. Therefore, an in‐depth study of the relationship between cognition and fractures may require a population with a broader baseline age, and analyses stratified by age, education (or literacy), or economic level while measuring cognition.

Because the CLSA is a national aging study and population‐based with a wide range of randomly recruited participants across Canada, selection bias due to the use of limited sample frames such as being recruited from single sites or recruiting only those over age 65 years was minimized. In addition, the results of this study can be generalized by using sample weights (ie, by assigning sample weights constructed based on inclusion probabilities) to each participant in the study so that the statistics computed from the data better represent the population. Moreover, this study assessed three domains of cognition: memory, executive function, and psychomotor speed by including available cognitive measures in CLSA. Thus, we could examine the relationship between multiple domains of cognition and fractures rather than evaluating only one domain in cognitive measures. Furthermore, to our knowledge, this study is the first study to simultaneously consider three factors (ie, cognition, frailty, and falls) related to fractures using a national population‐based aging study in Canada.

This study had several limitations. First, we were not sure if self‐reported incident fractures occurred between 2015 and 2017 because only self‐reported incident fractures between 2014 and 2015 from baseline and between 2017 and 2018 from 3‐year follow‐up were available. Second, as data on falls and fractures except for DXA femoral neck *T*‐score were from self‐report, there could be recall bias in completing the questionnaire. However, participants were asked about events during the last 12 months that would mitigate the effect of recall bias. Caution should be taken in interpreting our results. Although, the recall was over relatively short duration, we are not able to complete rule out recall bias. Third, fracture was only assessed through self‐reported questionnaires. The ascertainment of fracture is generally based on self‐reported with follow‐up confirmation by medical records (eg, radiographs, computed tomography [CT] scans, or surgical report) in population‐based studies of osteoporosis.^(^ ^53^ ^)^ However, we could not prove validity of self‐reported fractures by comparing later medical records. Previous studies have shown that the false positive (ie, “overreporting” that means fractures that did not occur)^(^ ^54^ ^)^ was 11%^(^ ^53^ ^)^ to 14.4%.^(^ ^54^ ^)^ Thus, it is expected that further future studies will need to be conducted to assess validity. Fourth, we included any fractures because we could not classify only major osteoporotic fractures (eg, hip, upper arm or shoulder, spine, or forearm) due to many missing values in fractures by skeletal sites. Fifth, our cohort was relatively young (ie, a mean age 59.5 years and with over 70% of the participants younger than age 65 years) with less cognitive impairment and frailty. However, we conducted a subgroup analysis of those aged 65 years or older that had 217 fractures, which revealed similar results that may lead to underpowering. In addition, only 0.2% and 1.6% of participants in our study responded they had dementia or Alzheimer's disease and memory problems, respectively. Cognitive impairment (especially at baseline) tends to be underrepresented in the CLSA because participants had to conduct the study on their own at baseline. Finally, there were more than 5% missing values in DXA femoral neck *T*‐score (ie, 7.4%) and household income (ie, 6.2%), which were variables that had the most missing values in the CLSA dataset. As we could not find any patterns in the missing data for those variables, we assumed the data were missing at random or completely at random, and thus conducted multiple imputation analysis. Any biases arising from the missing data may be toward the null.

In conclusion, frailty was independently associated with self‐reported incident fractures in both participants of all ages and in those aged 65 years or older, whereas a history of one or more falls was independently associated with self‐reported fractures in participants of all ages. In addition, the RAVLT: immediate recall test was associated with self‐reported incident fractures in participants of all ages and the MPMT was associated with fractures in those aged 65 years or older. No other performance measures were associated with self‐reported incident fractures, so it is possible that the associations between RAVLT: immediate recall test or MPMT and fractures are spurious. The mean value of the cognitive performance among participants with and without self‐reported incident fractures was similar. Thus, our results can also provide the basis for future studies to examine if adding frailty and a history of falls increases the predictive accuracy of current fracture risk assessment tools (ie, FRAX) and for nonpharmacological interventions for fracture prevention (eg, rehabilitation, fall prevention, and cognitive training).

## Author Contributions

**Ahreum Lee:** Conceptualization; data curation; formal analysis; investigation; methodology; project administration; resources; software; validation; writing – original draft; writing – review and editing. **Caitlin McArthur:** Conceptualization; data curation; funding acquisition; investigation; methodology; validation; writing – original draft; writing – review and editing. **George Ioannidis:** Conceptualization; investigation; methodology; validation; writing – original draft; writing – review and editing. **Jonathan D Adachi:** Conceptualization; investigation; validation; writing – review and editing. **Lauren E Griffith:** Conceptualization; investigation; validation; writing – review and editing. **Lehana Thabane:** Conceptualization; investigation; validation; writing – review and editing. **Lora M Giangregorio:** Conceptualization; investigation; validation; writing – review and editing. **Suzanne Nicole Morin:** Conceptualization; investigation; validation; writing – review and editing. **William Donald Leslie:** Conceptualization; investigation; validation; writing – review and editing. **Justin Lee:** Writing – review and editing. **Alexandra Papaioannou:** Conceptualization; data curation; funding acquisition; investigation; methodology; resources; supervision; validation; writing – original draft; writing – review and editing.

## Conflicts of Interest

All authors state that they have no conflicts of interest.

### Peer Review

The peer review history for this article is available at https://publons.com/publon/10.1002/jbm4.10679.

## Supporting information


**Table S1.** Participants’ baseline characteristics and comparisons between participants with and without self‐reported new fractures in the last year at follow‐up: (non‐weighted case)
**Table S2.** Results for sensitivity analysis in participants of all ages
**Table S3.** Results for sensitivity analysis in those aged over 65
**Table S4.** Results for multicollinearity tests of independent variables in participants of all ages
**Table S5.** Results for multicollinearity tests of independent variables in participants over 65

## Data Availability

Data are available from the Canadian Longitudinal Study on Aging (www.clsa-elcv.ca) for researchers who meet the criteria for access to de‐identified CLSA data.
