# Supplementary material for: Association Among Cognition, Frailty, and Falls and Self‐Reported Incident Fractures: Results From the Canadian Longitudinal Study on Aging (CLSA)
Source: JBMR Plus. 2022 Sep 28;6(10):e10679. doi: 10.1002/jbm4.10679 (PMC9549720; doi:10.1002/jbm4.10679)
Supplement: Supplementary file 1 — Table S1. Participants’ baseline characteristics and comparisons between participants with and without self‐reported new fractures in the last year at follow‐up: (non‐weighted case) Table S2. Results for sensitivity analysis in participants of all ages Table S3. Results for sensitivity analysis in those aged over 65 Table S4. Results for multicollinearity tests of independent variables in participants of all ages Table S5. Results for multicollinearity tests of independent variables in participants over 65 [file JBM4-6-e10679-s001.docx]

**Supplemental Tables**

Table S1. Participants’ baseline characteristics and comparisons between participants with and without self-reported new fractures in the last year at follow-up: (non-weighted case)

| Characteristics | All participants (*n* = 26,982) | Participants with self-reported fractures | | |
| --- | --- | --- | --- | --- |
|  |  | Yes  (*n* = 739) | No  (*n* = 26,243) | Standardized Difference^a^ |
| Age (years), mean (SD)  Age (years) group, n (%)  45 to 54  55 to 64  65 to 74  ≥75  Female sex, n (%)  BMI (kg/m^2^), mean (SD)  BMI (kg/m^2^) group, n (%)  Underweight (<18.5 kg/m^2^)  Normal weight (18.5 kg/m^2^ to 24.9 kg/m^2)^  Overweight (25.0 kg/m^2^ to 29.9 kg/m^2^)  Obese (≥30.0 kg/m^2^)  Ethnicity, n (%)  White  Non-white  Education, n (%)  Less than secondary school graduation  Secondary school graduation, no post-secondary education  Some post-secondary education  Post-secondary degree/diploma  Household income in past 12 months, n (%)  <$20,000  $20,000 to $49,999  $50,000 to $99,999  $100,000 to $149,999  ≥$150,000  Marital status, n (%)  Single, Never married  Married  Widowed, Divorced, Separated  Smoking status, n (%)  Non-smoker  Past smoker  Current smoker  Alcohol consumption in past 12 months, n (%)  Never  Less than once a month  1 to 4 times a month  2 to 5 times a week  Almost every day  Parental hip fracture history, n (%)  Previous fractures during adult life, n (%)  Corticosteroids use, n (%)  Self-reported rheumatoid arthritis status, n (%)  Self-reported osteoporosis status, n (%)  DXA femoral neck T-score, mean (SD)  Osteoporosis category based on WHO classification, n (%)  Normal (T-score≥-1)  Osteopenia (-2.5<T-score<-1)  Osteoporosis (T-score≤-2.5)  Cognitive measures, mean (SD)  RAVIT - Immediate Recall  RAVIT - 5 min Delayed Recall  Animal Fluency Test - Strict  MAT  MPMT - Total  Stroop Test - Interference Ratio (Color/Dot)^b^  COWAT - Total  CRT - Mean Response Time (ms)^b^  Frailty Index, mean (SD)  History of falls n (%)  Non-faller (no)  Faller (yes) | 62.7 (10.1)  6,946 (25.7)  8,988 (33.3)  6,610 (24.5)  4,438 (16.5)  13,648 (50.6)  28.05 (5.4)  177 (0.7)  7,984 (29.7)  10,864 (40.4)  7,855 (29.2)  22,660 (84.0)  4,322 (16.0)  1,335 (5.0)  2,474 (9.2)  1,953 (7.3)  21,178 (78.6)  1,250 (4.9)  5,475 (21.6)  9,028 (35.7)  5,086 (20.1)  4.478 (17.7)  2,114 (8.1)  18,117 (69.3)  5,927 (22.6)  12,967 (48.1)  11,742 (43.5)  2,272 (8.4)  2,910 (11.0)  3,225 (12.2)  7,478 (28.4)  8,372 (31.8)  4,376 (16.6)  3,458 (13.0)  3,770 (14.0)  3,487 (13.2)  843 (3.2)  2,301 (8.6)  -0.68 (1.2)  14,957 (55.4)  8,875 (32.9)  3,150 (11.7)  5.9 (1.9)  4.1 (2.2)  19.9 (5.6)  26.8 (8.7)  17.1 (2.0)  2.2 (0.7)  39.3 (12.8)  845.5 (238.5)  0.118 (0.08)  26,033 (96.5)  948 (3.5) | 63.5 (10.6)  178 (24.1)  241 (32.6)  164 (22.2)  156 (21.1)  480 (65.0)  27.55 (5.1)  3 (0.4)  237 (32.1)  324 (43.8)  175 (23.7)  595 (80.5)  144 (19.5)  37 (5.0)  73 (9.9)  69 (9.4)  559 (75.8)  43 (6.3)  179 (26.3)  224 (32.9)  115 (16.9)  119 (17.5)  52 (7.2)  447 (62.0)  222 (30.8)  339 (45.9)  330 (44.7)  70 (9.5)  73 (10.1)  95 (13.2)  203 (28.2)  218 (30.2)  132 (18.3)  121 (16.6)  173 (23.4)  117 (16.1)  46 (6.3)  111 (15.2)  -1.07 (1.1)  313 (42.4)  301 (40.7)  125 (16.9)  6.1 (2.0)  4.3 (2.2)  19.9 (5.6)  26.3 (8.8)  17.0 (2.0)  2.2 (0.7)  39.9 (12.87)  858.0 (237.7)  0.135 (0.086)  697 (94.3)  42 (5.7) | 62.7 (10.1)  6,768 (25.8)  8,747 (33.3)  6,446 (24.6)  4,282 (16.3)  13,168 (50.2)  28.06 (5.4)  174 (0.7)  7,747 (29.6)  10,540 (40.3)  7,680 (29.4)  22,065 (84.1)  4,178 (15.9)  1,298 (5.0)  2,401 (9.2)  1,884 (7.2)  20,619 (78.7)  1,207 (4.9)  5,296 (21.5)  8,804 (35.7)  4,971 (20.2)  4,359 (17.7)  2,062 (8.1)  17,730 (69.5)  5,705 (22.4)  12,628 (48.1)  11,412 (43.5)  2,202 (8.4)  2,837 (11.1)  3,130 (12.2)  7,275 (28.4)  8,154 (31.8)  4,244 (16.6)  3,337 (12.9)  3,597 (13.8)  3,370 (13.1)  797 (3.1)  2,190 (8.4)  -0.66 (1.2)  14,644 (55.8)  8,574 (32.7)  3,025 (11.5)  5.9 (1.9)  4.1 (2.2)  19.9 (5.6)  26.9 (8.7)  17.1 (2.0)  2.1 (0.7)  39.3 (12.8)  845.2 (238.5)  0.118 (0.076)  25,336 (96.6)  906 (3.5) | 0.072  0.123  -0.302  -0.093  -0.127  -0.094  -0.069  -0.014  0.190  0.038  0.045  0.105  0.251  0.083  0.154  0.211  -0.348  0.168  0.077  0.084  -0.004  -0.074  -0.045  -0.087  -0.080  0.195  0.107 |

BMI = Body Mass Index; RAVLT = Rey Auditory Verbal Learning Test (range from 0 to 15); Animal Fluency Test (range from 0 to 52); MAT = Mental Alternation Test (range from 0 to 52); MPMT = Miami Prospective Memory Test (range from 0 to 18); COWAT = Controlled Oral Word Association Test (range from 3 to 105); CRT = Choice Reaction Time (range from 79 to 9,958)

^a^Standardized difference is difference in means or proportions divided by standard error. Imbalance defined as absolute value greater than 0.20 (small effect size).

^b^Lower values reflect better performance.

Table S2. Results for sensitivity analysis in participants of all ages

|  | Adjusted OR^a^ (95% confidence interval) | *p* Value |
| --- | --- | --- |
| *Complete case analysis (non-weighted case)*  Cognitive measures  RAVLT - Immediate Recall [per-1.89 (one SD) increment]  RAVLT - 5 min Delayed Recall [per-2.16 (one SD) increment]  Animal Fluency Test - Strict [per-5.64 (one SD) increment]  MAT [per-8.65 (one SD) increment]  MPMT [per-1.98 (one SD) increment]  COWAT [per-12.84 (one SD) increment]  Stroop Test - Interference Ratio (color/dot) [per-0.71 (one SD) increment]  CRT - Mean Response Time [per-238.52 (one SD) increment]  Frailty Index [per-0.08 (one SD) increment]  History of falls  Non-faller (no)  Faller (yes)  *Complete case analysis (weighted case)*  Cognitive measures  RAVLT - Immediate Recall [per-1.89 (one SD) increment]  RAVLT - 5 min Delayed Recall [per-2.16 (one SD) increment]  Animal Fluency Test - Strict [per-5.64 (one SD) increment]  MAT [per-8.65 (one SD) increment]  MPMT [per-1.98 (one SD) increment]  COWAT [per-12.84 (one SD) increment]  Stroop Test - Interference Ratio (color/dot) [per-0.71 (one SD) increment]  CRT - Mean Response Time [per-238.52 (one SD) increment]  Frailty Index [per-0.08 (one SD) increment]  History of falls  Non-faller (no)  Faller (yes) | 1.08 (0.94-1.23)  0.99 (0.87-1.13)  1.09 (0.98-1.22)  0.98 (0.88-1.09)  1.05 (0.95-1.17)  1.03 (0.93-1.14)  0.97 (0.89-1.05)  0.95 (0.87-1.04)  1.21 (1.09-1.34)  Reference  1.61 (1.10-2.36)  1.11 (0.94-1.30)^b^  0.99 (0.82-1.20)^b^  1.10 (0.95-1.27)^b^  1.05 (0.90-1.22)^b^  1.07 (0.94-1.22)^b^  1.00 (0.85-1.13)^b^  1.00 (0.91-1.11)^b^  1.02 (0.89-1.17)^b^  1.25 (1.07-1.48)^b^  Reference  1.81 (0.99-3.31)^b^ | .275  .886  .114  .678  .336  .627  .441  .239  <.001  .015  .224  .916  .199  .569  .334  .739  .943  .779  .006  .053 |

RAVLT = Rey Auditory Verbal Learning Test; MAT = Mental Alternation Test; MPMT = Miami Prospective Memory Test; COWAT = Controlled Oral Word Association Test; CRT = Choice Reaction Time

^a^Adjusted for age, sex, ethnicity, educational level, marital status, income, smoking, alcohol consumption, BMI group, parental hip fracture history, prior fracture, corticosteroids use, self-reported rheumatoid arthritis status, self-reported osteoporosis status and DXA femoral neck T-score

^b^95% confidence limits

Table S3. Results for sensitivity analysis in those aged over 65

|  | Adjusted OR^a^ (95% confidence interval) | *p* Value |
| --- | --- | --- |
| *Complete case analysis (non-weighted case)*  Cognitive measures  RAVLT - Immediate Recall [per-1.79 (one SD) increment]  RAVLT - 5 min Delayed Recall [per-1.98 (one SD) increment]  Animal Fluency Test - Strict [per-5.15 (one SD) increment]  MAT [per-8.53 (one SD) increment]  MPMT [per-2.41 (one SD) increment]  COWAT [per-12.89 (one SD) increment]  Stroop Test - Interference Ratio (color/dot) [per-0.77 (one SD) increment]  CRT - Mean Response Time [per-271.94 (one SD) increment]  Frailty Index [per-0.08 (one SD) increment]  History of falls  Non-faller (no)  Faller (yes)  *Complete case analysis (weighted case)*  Cognitive measures  RAVLT - Immediate Recall [per-1.79 (one SD) increment]  RAVLT - 5 min Delayed Recall [per-1.98 (one SD) increment]  Animal Fluency Test - Strict [per-5.15 (one SD) increment]  MAT [per-8.53 (one SD) increment]  MPMT [per-2.41 (one SD) increment]  COWAT [per-12.89 (one SD) increment]  Stroop Test - Interference Ratio (color/dot) [per-0.77 (one SD) increment]  CRT - Mean Response Time [per-271.94 (one SD) increment]  Frailty Index [per-0.08 (one SD) increment]  History of falls  Non-faller (no)  Faller (yes) | 1.09 (0.96-1.23)  0.91 (0.82-1.01)  1.02 (0.99-1.06)  1.01 (0.99-1.03)  1.03 (0.95-1.12)  1.00 (0.99-1.02)  1.05 (0.82-1.34)  1.00 (1.00-1.00)  1.31 (1.10-1.55)  Reference  2.40 (1.33-4.31)  1.11 (0.95-1.31)^b^  0.95 (0.82-1.09)^b^  1.02 (0.98-1.06)^b^  1.00 (0.97-1.03)^b^  1.02 (0.93-1.11)^b^  1.00 (0.98-1.02)^b^  1.11 (0.81-1.51)^b^  1.00 (1.00-1.00)^b^  1.40 (1.12-1.74)^b^  Reference  1.87 (0.95-3.69)^b^ | .187  .085  .237  .352  .431  .954  .705  .383  .002  .004  .199  .435  .296  .977  .707  .815  .518  .952  .003  .071 |

RAVLT = Rey Auditory Verbal Learning Test; MAT = Mental Alternation Test; MPMT = Miami Prospective Memory Test; COWAT = Controlled Oral Word Association Test; CRT = Choice Reaction Time

^a^Adjusted for age, sex, ethnicity, educational level, marital status, income, smoking, alcohol consumption, BMI group, parental hip fracture history, prior fracture, corticosteroids use, self-reported rheumatoid arthritis status, self-reported osteoporosis status and DXA femoral neck T-score

^b^95% confidence limits

Table S4. Results for multicollinearity tests of independent variables in participants of all ages

| Multicollinearity of independent variables | VIF |
| --- | --- |
| RAVLT – Immediate Recall  RAVLT – 5 min Delayed Recall  Animal Fluency Test - Strict  MAT  MPMT – Total  COWAT – Total  Stroop Test – Interference Ratio (color/dot)  CRT – Mean Response Time  Frailty Index  A history of falls | 2.133  2.073  1.470  1.407  2.580  1.403  5.168  1.176  1.386  1.004 |

VIF = Variance Inflation; RAVLT = Rey Auditory Verbal Learning Test; MAT = Mental Alternation Test; MPMT = Miami Prospective Memory Test; COWAT = Controlled Oral Word Association Test; CRT = Choice Reaction Time

Table S5. Results for multicollinearity tests of independent variables in participants over 65

| Multicollinearity of independent variables | VIF |
| --- | --- |
| RAVLT – Immediate Recall  RAVLT – 5 min Delayed Recall  Animal Fluency Test - Strict  MAT  MPMT – Total  COWAT – Total  Stroop Test – Interference Ratio (color/dot)  CRT – Mean Response Time  Frailty Index  A history of falls | 1.951  1.875  1.365  1.300  1.098  1.374  1.041  1.068  1.321  1.005 |

VIF = Variance Inflation; RAVLT = Rey Auditory Verbal Learning Test; MAT = Mental Alternation Test; MPMT = Miami Prospective Memory Test; COWAT = Controlled Oral Word Association Test; CRT = Choice Reaction Time
